# Effect of annealing on the magnetic microstructure of high-pressure torsion iron: the relevance of higher-order contributions to the magnetic small-angle neutron scattering cross section

**DOI:** 10.1107/S2052252523003937

**Published:** 2023-05-19

**Authors:** Mathias Bersweiler, Hirokazu Sato, Nozomu Adachi, Yoshikazu Todaka, Inma Peral, Joachim Kohlbrecher, Vladislav D. Zaporozhets, Konstantin L. Metlov, Andreas Michels, Yojiro Oba

**Affiliations:** a Department of Physics and Materials Science, Université du Luxembourg, 162A avenue de la Faïencerie, Luxembourg L-1511, Grand Duchy of Luxembourg; b Department of Mechanical Engineering, Toyohashi University of Technology, 1-1 Hibarigaoka, Tempaku, Toyohashi, Aichi 441-8580, Japan; c Laboratory for Neutron Scattering, ETH Zurich & Paul Scherrer Institut, Villigen, PSI 5232, Switzerland; d Donetsk Institute for Physics and Technology, R. Luxembourg Street 72, Donetsk 83114, Ukraine; e Institute for Numerical Mathematics RAS, 8 Gubkina Street, Moscow GSP-1 119991, Russian Federation; f Materials Sciences Research Center, Japan Atomic Energy Agency, 2-4 Shirakata, Tokai, Ibaraki 319-1195, Japan; Australian Nuclear Science and Technology Organisation and University of Wollongong, Australia

**Keywords:** small-angle neutron scattering, magnetism, high-pressure torsion, nanocrystalline materials, materials science, magnetic scattering

## Abstract

The development of higher-order micromagnetic small-angle neutron scattering theory in nanocrystalline materials is still in its infancy. One key challenge remaining in this field is understanding the role played by the microstructure on the magnitude and sign of the higher-order scattering contribution recently observed in nanocrystalline materials prepared by high-pressure torsion. By combining structural and magnetic characterization techniques, namely X-ray diffraction, electron backscattered diffraction and magnetometry with magnetic small-angle neutron scattering, this work discusses the relevance of higher-order terms in the magnetic small-angle neutron scattering cross section of pure iron prepared by high-pressure torsion associated with a post-annealing process. The structural analysis confirms: (i) the preparation of ultra-fine-grained pure iron with a crystallite size below 100 nm and (ii) rapid grain growth with increasing annealing temperature. The analysis of neutron data based on the micromagnetic small-angle neutron scattering theory extended to textured ferromagnets yields uniaxial magnetic anisotropy values that are larger than the magnetocrystalline value reported for bulk iron, supporting the existence of induced magnetoelastic anisotropy in the mechanically deformed samples. Furthermore, the neutron data analysis revealed unambiguously the presence of non-negligible higher-order scattering contributions in high-pressure torsion iron. Though the sign of the higher-order contribution might be related to the amplitude of the anisotropy inhomogeneities, its magnitude appears to be clearly correlated to the changes in the microstructure (density and/or shape of the defects) induced by combining high-pressure torsion and a post-annealing treatment.

## Abstract 2

The role of higher-order small-angle neutron scattering effects in ultra-fine-grained pure iron prepared by combining high-pressure torsion with a post-annealing process is investigated.

## 1. Introduction

High-pressure torsion (HPT) is a well known and efficient severe plastic deformation technique in the materials science community (Valiev *et al.*, 2006 ▸; Edalati & Horita, 2016 ▸; Horita & Edalati, 2020 ▸; Edalati *et al.*, 2022 ▸); it is used to introduce a significant grain refinement and a high density of lattice defects in bulk nanocrystalline materials. For instance, Adachi *et al.* (2018 ▸) investigated the mechanical properties of ultra-fine-grained pure iron (Fe) fabricated by HPT through tensile testing and *in situ* neutron diffraction. These authors have found that as-prepared HPT Fe exhibits a large uniform elongation relative to annealed Fe and that it accommodates plastic strain uniformly irrespective of the grain orientation. Although the characteristic microstructures produced by HPT can improve the mechanical properties, a considerable effort is devoted to understanding the effect of HPT on the magnetic properties. HPT already represents a promising approach to control the macroscopic magnetic properties of metals, in particular it has become possible to induce ferromagnetism in paramagnetic metals (Cepeda-Jiménez *et al.*, 2016 ▸, 2017 ▸) or to improve their soft-magnetic character (Scheriau *et al.*, 2010 ▸). More recently, magnetic small-angle neutron scattering (SANS) experiments reported a significant increase of the effective magnetic anisotropy in HPT Fe (Oba *et al.*, 2020 ▸) and HPT Ni (Bersweiler *et al.*, 2021 ▸; Zaporozhets *et al.*, 2022 ▸), opening up a new route for the development of advanced magnetic materials using severe plastic-deformation techniques (Horita & Edalati, 2020 ▸). However, the analysis of the magnetic SANS cross section in HPT Ni also revealed an unusual, predominant longitudinal sine-squared-type angular anisotropy, which is beyond the classical second-order micromagnetic SANS theory and can be qualitatively understood by the higher-order theory (Oba *et al.*, 2021 ▸). A quantitative analysis of such a dominant (negative) higher-order scattering contribution was not performed to date and even its origin in nanocrystalline HPT Ni remains unclear.

The purpose of the present study is (i) to quantify the ‘anomalous’ magnetoelastic anisotropy previously suggested in pure HPT Fe and (ii) to further contribute to the understanding of the role played by the microstructure in the observation of higher-order contributions to the magnetic SANS cross section of HPT materials. In particular, we focus our analysis on the grain-size dependence of the structural and magnetic properties of nanocrystalline HPT Fe using a combination of standard techniques (*i.e.* X-ray diffraction, electron backscattered diffraction and magnetometry) with unpolarized magnetic SANS. The latter method is very powerful for obtaining bulk-averaged information on mesoscale structural and magnetic inhomogeneities; more specifically, magnetic SANS provides information about the variation of the magnetization vector field on a length scale of about 1–300 nm (Mühlbauer *et al.*, 2019 ▸; Michels, 2021 ▸). For instance, magnetic SANS was previously employed to probe the spin structure of Heusler alloys (Runov *et al.*, 2006 ▸; Bhatti *et al.*, 2012 ▸; El-Khatib *et al.*, 2019 ▸; McCalla *et al.*, 2021 ▸; Bersweiler *et al.*, 2022*b* ▸), magnetic nanocomposites (Ito *et al.*, 2007 ▸; Bick *et al.*, 2013 ▸) or more recently to unravel the magnetic softness of Fe–Ni–B alloy, a HiB-NANOPERM-type soft magnetic material (Bersweiler *et al.*, 2022*a* ▸).

The paper is organized as follows: Section 2 provides details about the HPT sample preparation, the structural and magnetic characterization, as well as the neutron experiments. Section 3 provides a brief overview of the main expressions for the unpolarized magnetic SANS cross section, the recently derived analytical expressions for the spin-misalignment SANS cross section of textured ferromagnets are displayed and the neutron-data analysis is sketched. Section 4 presents and discusses the experimental results, and Section 5 summarizes the main findings of this study.

## 2. Experimental

The procedure for the preparation of the HPT Fe samples used in this paper is similar to the one described by Adachi *et al.* (2018 ▸). Sheets of Fe were cut into circular disks with a diameter of 20 mm. Prior to the HPT process, the samples were annealed at 1073 K for 1 h to homogenize the microstructure. The HPT process was then conducted under a compressive pressure of 5 GPa for *N* = 10 turns and with a rotation speed of 0.2 rpm. Subsequently, the disk-shaped samples were polished to remove surface roughness, oxides and possible impurities and were annealed at 473, 673 and 1273 K for 1 h; these are typical annealing temperatures for HTP Fe (Todaka *et al.*, 2008 ▸; Shugaev *et al.*, 2022 ▸). The final thickness of all the HPT samples was 0.36 mm. As previously reported, this specific approach of combining the HPT technique with an annealing process is a very effective way to easily obtain ultra-fine-grained Fe samples with various grain sizes (Adachi *et al.*, 2018 ▸). In the following, the samples will be labeled as HPT + *T*_a_, where *T*_a_ denotes the annealing temperature. Disks of non-deformed (nd) Fe with a diameter of 20 mm and a thickness of 0.88 mm were also prepared for comparison. Note that the thickness difference between the HPT + *T*_a_ samples and the nd Fe sample results from the polishing after the HPT process and has no influence on the magnetic SANS results discussed below.

The microstructure of the HPT and nd Fe samples was characterized by wide-angle X-ray diffraction (XRD) using a Bruker D8 DISCOVER diffractometer in Bragg–Brentano geometry (Cu *K*α radiation) and by electron backscattered diffraction (EBSD) using a Schottky field emission scanning electron microscope (SU5000, Hitachi High-Tech Corporation) with an electron backscatter detector (EDAX, Inc.). The EBSD patterns obtained were analyzed by the *OIM* analysis software (EDAX, USA). To fit with the requirements of the magnetometry and neutron experiments, rectangular-shaped samples with the surface area 10 × 4 mm were cut. Room-temperature magnetization curves for all samples were recorded using a Cryogenic Ltd vibrating sample magnetometer equipped with a 14 T superconducting magnet.

**Figure 1:**
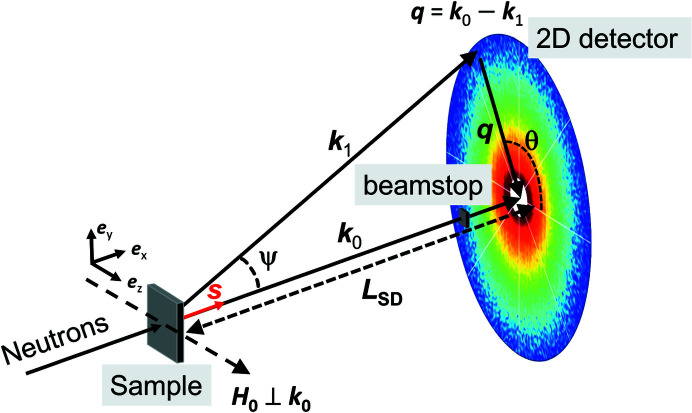
Sketch of the magnetic SANS geometry. The momentum-transfer vector **q** corresponds to the difference between the wavevectors of the incident (**k**_0_) and scattered (**k**_1_) neutrons, *i.e.* **q** = **k**_0_ − **k**_1_. The magnetic field **H**_0_ is applied perpendicular to the incident neutron beam, *i.e.* 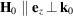 . The vector **s** represents the texture-axis direction, which in this study is parallel to the incident neutron beam, *i.e.* **k**_0_. The azimuthal angle θ characterizes the angular anisotropy of the recorded scattering pattern on the 2D detector. *L*_SD_ corresponds to the sample-to-detector distance. For small-angle scattering, the component of the scattering vector along the incident neutron beam, here *q_x_*, is generally much smaller than the other two components *q_y_* and *q_z_*, so that only correlations in the plane perpendicular to the incoming neutron beam are probed.

The neutron experiments were conducted at the instrument SANS-1 at the Swiss Spallation Neutron Source at the Paul Scherrer Institute, Switzerland. Fig. 1 ▸ is a sketch of the experimental SANS setup used for this study. The measurements were carried out using an unpolarized incident neutron beam with a mean wavelength of λ = 6.0 Å and a wavelength broadening of Δλ/λ = 10% (full width at half-maximum). Sample-to-detector distances (*L*_SD_) of 18 and 4.5 m were chosen to cover a *q* range of about 0.033 nm^−1^ ≤ *q* ≤ 0.94 nm^−1^ [*q* = (4π/λ)/sin(Ψ/2), where Ψ is the scattering angle and λ is the wavelength of the incident radiation]. A magnetic field **H**_0_ was applied perpendicular to the incident neutron beam (**H**_0_ ⊥ **k**_0_). We would like to emphasize that, in this geometry, **H**_0_ is perpendicular to the texture axis **s** that is induced by the HPT process (**H**_0_ 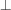 **s**). Neutron data were recorded by decreasing the field from the maximum field available of 8.0 T down to 0.2 T, following the magnetization curve (see Fig. 3). Within this field range, the neutron transmission values were larger than 90%, indicating negligible multiple-scattering contributions. The neutron data reduction (corrections for background scattering and sample transmission) was conducted using the *GRASP* software package (Dewhurst, 2023 ▸).

## 3. Micromagnetic SANS theory

Based on the micromagnetic SANS theory for inhomogeneous ferromagnets (Honecker & Michels, 2013 ▸), the elastic total (nuclear + magnetic) unpolarized SANS cross section dΣ/dΩ at momentum-transfer vector **q** can be formally written as

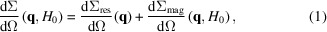

where, for the perpendicular scattering geometry (**H**_0_ ⊥ **k**_0_), the first term

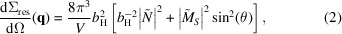

represents the residual SANS cross section, which is measured at complete magnetic saturation, and the second term

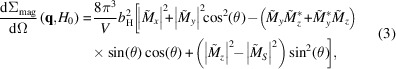

corresponds to the purely magnetic SANS cross section, which disappears at saturation. In the above expressions, *V* is the scattering volume, *b*_H_ = 2.91 × 10^8^ A^−1^ m^−1^ is the atomic magnetic scattering length in the small-angle regime, 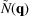 and 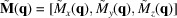 represent the Fourier transforms of the nuclear scattering length density *N*(***r***) and of the magnetization vector field **M**(**r**), respectively, and ‘*’ denotes the complex conjugated quantities. *M_S_* (**q**) denotes the Fourier transform of the saturation magnetization profile *M_S_* (**r**).

In the neutron data analysis (see Section 4), to experimentally access the 2D dΣ_mag_/dΩ, we subtracted the 2D total SANS cross section dΣ/dΩ measured at the largest field of 8 T (approach-to-saturation regime; compare Fig. 3) from those measured at lower fields. This specific subtraction procedure eliminates the nuclear SANS contribution 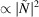 , which is field independent, and therefore leads to the following expression for dΣ_mag_/dΩ:

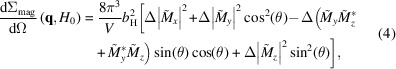

where Δ represents the differences of the Fourier components at the two selected fields (low field − highest field).

In the case of the approach-to-saturation regime, where 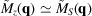 , the second-order micromagnetic SANS theory rigorously predicts that 
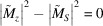
 , so that the fourth term 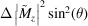 in equation (4) disappears. Using the analytical results for the magnetization Fourier components of Honecker & Michels (2013 ▸) and Zaporozhets *et al.* (2022 ▸), it becomes possible to re-write equation (4) as

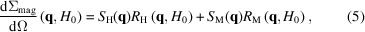

where *S*_H_ *R*_H_ and *S*_M_ *R*_M_ correspond to the magnetic scattering contributions due to perturbing magnetic anisotropy fields and magnetostatic fields, respectively. In the particular case of an inhomogeneous polycrystalline ferromagnet with a global uniaxial anisotropy (magnetic texture), and when the texture axis is parallel to the incident neutron beam (as in our study), the anisotropy-field scattering function *S*_H_(*q*), the scattering function of the longitudinal magnetization *S*_M_(*q*), and the corresponding micromagnetic response functions *R*_H_(*q*, θ) and *R*_M_(*q*, θ) are given as follows (Zaporozhets *et al.*, 2022 ▸):

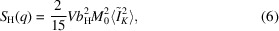




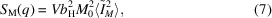




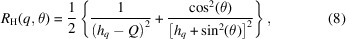




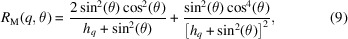

where 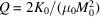 is the global anisotropy quality factor and *K*_0_ (in J m^−3^) denotes the strength of the effective anisotropy, 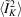 corresponds to the average of the squared Fourier image of the anisotropy constant fluctuations, 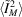 is the corresponding quantity for the saturation magnetization fluctuations (assumed to be of the same order of magnitude as 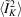 ), *M*_0_ = 〈*M_S_* (**r**)〉 represents the average saturation magnetization, and 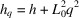 is a dimensionless quantity related to the dimensionless applied-field parameter *h* = *H*_0_/*M*_0_ and to the exchange length 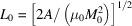 with the exchange-stiffness constant *A*. Equations (5)–(9) have already been successfully used to fit the angular dependence of the spin-misalignment SANS cross section of nanocrystalline HPT Ni, allowing one to estimate the global uniaxial anisotropy constant (Zaporozhets *et al.*, 2022 ▸).

## 4. Results and discussion

**Figure 2:**
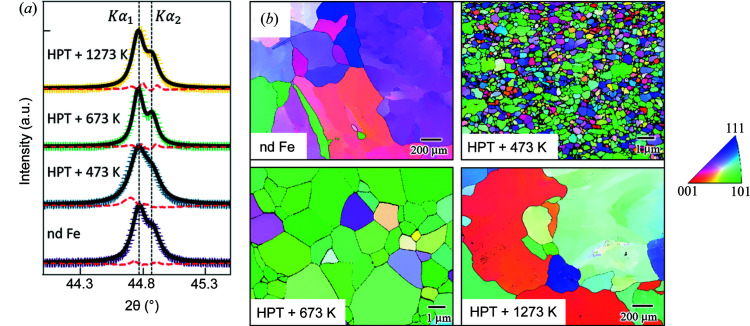
(*a*) XRD patterns of the annealed HPT Fe specimens and the nd Fe reference sample in the region of the (110) reflection (Cu *K*α radiation). The data have been normalized to the maximum of the (110) peak and vertically shifted for clarity. Black solid lines: XRD data refinement using the Le Bail fit method implemented in the open-source software *FullProf* (Rodríguez-Carvajal, 1993 ▸). Red dashed lines represent the difference between the calculated and measured intensities. (*b*) Inverse pole figure maps of HPT Fe and nd Fe obtained using EBSD. Uniformly colored areas represent regions of the same (or nearly the same) crystallographic orientation.

Fig. 2 ▸(*a*) shows wide-angle X-ray diffraction data in the region of the (110) reflection for the annealed HPT Fe samples and for the nd Fe reference. We can see that although the (110) diffraction peak for HPT + 473 K is broad and not separable, the *K*α_1_ and *K*α_2_ lines for the (110) Fe reflection start to be resolved for HPT + 673 K and HPT + 1273 K. These features are indicative of an increase of the average crystallite size after the annealing process in our HPT samples. For the estimation of the average crystallite size *D*_XRD_, the Le Bail fit method implemented in the open-source software suite *FullProf* (Rodríguez-Carvajal, 1993 ▸) was used (considering the space group *Im* 3 *m*). The best-fit values are summarized in Table 1 ▸. The two following features are observed: (i) an ultra-fine-grained microstructure with *D*_XRD_ < 100 nm is obtained; and (ii) *D*_XRD_ increases with increasing annealing temperature *T*_a_ up to 673 K, before it slightly decreases again at 1273 K. Fig. 2 ▸(*b*) presents the inverse pole figure maps of HPT Fe and nd Fe obtained using EBSD. The estimated average grain sizes *D*_EBSD_ are summarized in Table 1 ▸. Note that here the term ‘grain’ refers to an aggregate of several crystallites with the same (or nearly the same) crystallographic orientation. The individual crystallites cannot clearly be observed in Fig. 2 ▸(*b*), probably because of the resolution limit of the EBSD analysis. In this way, the rapid grain growth with increasing *T*_a_ becomes more clearly visible. Furthermore, the difference between *D*_XRD_ and *D*_EBSD_ (see Table 1 ▸) suggests that the number of crystallites in a grain increases significantly with increasing *T*_a_. This result is related to recrystallization and subsequent grain growth, which occur for annealing temperatures higher than 473 K. As discussed in many publications, modified and enhanced recrystallization by HPT straining is the driving force of the grain-size dependence with the annealing temperature in HPT Fe (Voronova *et al.*, 2007 ▸; Oberdorfer *et al.*, 2010 ▸). Moreover, it is well known that the recrystallization process also gives rise to a recrystallization texture of an axial 〈110〉 type in HPT Fe (Shugaev *et al.*, 2022 ▸). In Fig. 2 ▸(*b*), for HPT + 673 K, this is clearly seen as the development of {110} planes. The smaller population of the grains probably makes the observation of this texture more difficult for HPT + 1273 K.

**Figure 3:**
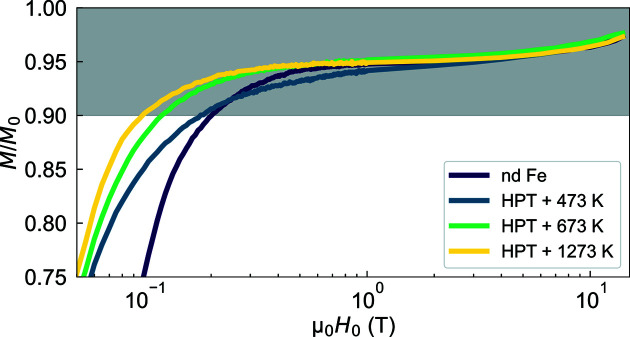
Upper-right branch of the normalized magnetizations of HPT Fe and nd Fe measured at room temperature and between + 14 T and + 0.05 T (semi-logarithmic scale). Gray area: approach-to-saturation regime defined as *M*/*M*_0_ ≥ 90%. The magnetization curves have been normalized by the saturation magnetizations *M*_0_, which were estimated for each sample from the linear regression *M*(1/*H*_0_) in the high-field regime (*i.e.* μ_0_ *H*_0_ 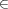 10 T − 14 T). The corresponding values are given in Table 1 ▸.

Fig. 3 ▸ displays the magnetization curves of HPT Fe and nd Fe on a semi-logarithmic scale. From the analysis of the magnetization curves, we estimated the saturation magnetizations *M*_0_ (see Table 1 ▸). As shown in Fig. 3 ▸, the magnetization curves exhibit no significant differences in the high-field regime. The HPT + 473 K sample reveals a μ_0_ *M*_0_ value that is close to the room-temperature value (2.15 T) of single crystalline Fe (Crangle & Goodman, 1971 ▸). The other specimens, including the nd Fe sample, exhibit reduced saturation magnetizations (by about 5%). Although the origin for the lower *M*_0_ of HPT + 673 K and HPT + 1273 K might be related to the HPT process [as previously reported for HPT Ni (Mulyukov *et al.*, 1992 ▸; Bersweiler *et al.*, 2021 ▸)], the reasons for the (low) value of 2.06 T of nd Fe and for the (high) value of 2.16 T for HPT + 473 K remain unknown. Defining the approach-to-saturation regime by *M*/*M*_0_ ≥ 90%, we can see that this regime is attained for μ_0_ *H*_0_ larger than about 0.2 T. This is important since the micromagnetic SANS data analysis is restricted to this high-field regime. Note that the (small) coercivity of the present samples cannot be reliably determined with our magnetometer; DC hysteresis loop tracer measurements are required for this.

**Figure 4:**
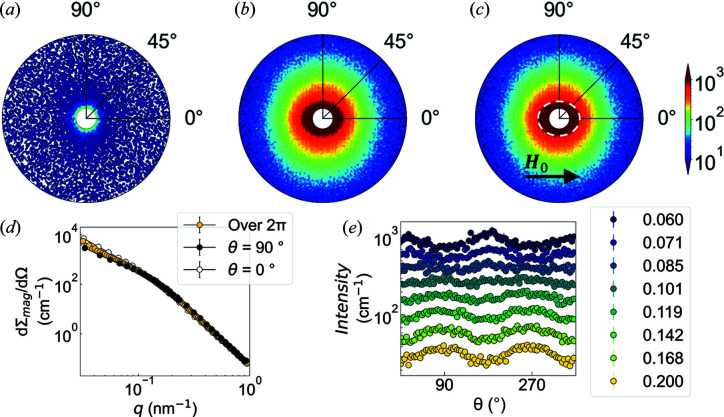
Experimental 2D elastic total (nuclear + magnetic) unpolarized SANS cross section dΣ/dΩ of the HPT + 473 K Fe sample measured at the selected fields of (*a*) 8 T and (*b*) 0.2 T. (*c*) The corresponding purely magnetic SANS cross section dΣ_mag_/dΩ obtained by subtracting the dΣ/dΩ at the (near) saturation field of 8.0 T from the dΣ/dΩ at μ_0_ *H*_0_ = 0.2 T. The applied magnetic field **H**_0_ is horizontal in the plane of the detector (**H**_0_ ⊥ **k**_0_) and a logarithmic color scale is used in (*a*)–(*c*). (*d*) Experimental 1D purely magnetic SANS cross section obtained either by an azimuthal average over 2π, or from ±10° sector averages along (θ = 0°) or perpendicular (θ = 90°) to **H**_0_ of the dΣ_mag_/dΩ plotted in (*c*) (log–log scale). (*e*) Azimuthal angle (θ) dependency of the magnetic scattering intensities obtained from (*c*). Note that in (*a*)–(*c*) dΣ/dΩ and dΣ_mag_/dΩ are plotted in polar coordinates with *q* (nm^−1^), θ (°) and intensity (cm^−1^). The data in (*a*)–(*c*) are shown up to *q* = 0.25 nm^−1^. The white dashed lines in (*c*) are a visual guide to highlight the slight elongation of the scattering pattern along the field direction at smaller *q*.

Figs. 4 ▸(*a*) and 4 ▸(*b*) display typical examples of the experimental 2D total SANS cross sections dΣ/dΩ of the HPT + 473 K Fe sample at the selected fields of 8 and 0.2 T, respectively. As can be seen, at μ_0_ *H*_0_ = 8 T (near saturation), dΣ/dΩ exhibits a nearly isotropic pattern, which suggests the dominance of isotropic nuclear scattering. According to the magnetization curves [see Fig. 3 ▸], the sample is nearly magnetically saturated at a magnetic field of 8 T. By reducing *H*_0_, the pattern at the larger (smaller) momentum transfers becomes slightly elongated perpendicular (parallel) to **H**_0_, suggesting a more complex magnetization structure corresponding to the other terms in the magnetic SANS cross section [equation (4)]. Fig. 4 ▸(*c*) shows the corresponding 2D purely magnetic SANS cross section dΣ_mag_/dΩ, obtained from the subtraction of (*a*) and (*b*). As is seen, the vertical and horizontal elongations remain in dΣ_mag_/dΩ in the respective *q* range after the subtraction procedure [compare also Figs. 4 ▸(*d*) and 4(*e*)]. The horizontal elongation can be attributed to the spin-misalignment scattering due to long-wavelength transversal magnetization fluctuations [compare the 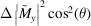 term in equation (4)]. As recently discussed by Oba *et al.* (2021 ▸) for ultra-fine-grained Ni prepared by HPT, the vertical elongation might be related to the higher-order terms in the expression for dΣ_mag_/dΩ [compare the 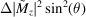 term in equation (4)]. These hypotheses are supported by the angular dependence of the magnetic scattering intensity shown in Fig. 4 ▸(*e*), where the two maxima centered at θ = 90° (0°) and at θ = 270° (180°) at the larger (smaller) momentum transfers are characteristic of a sine (cosine) squared function. Note that a similar observation was made by Mettus *et al.* (2017 ▸), who studied a series of as-cast, aged and mechanically deformed bulk metallic glasses. The relevance of the higher-order terms in the magnetic SANS cross section will be discussed more in detail later in the manuscript.

**Figure 5:**
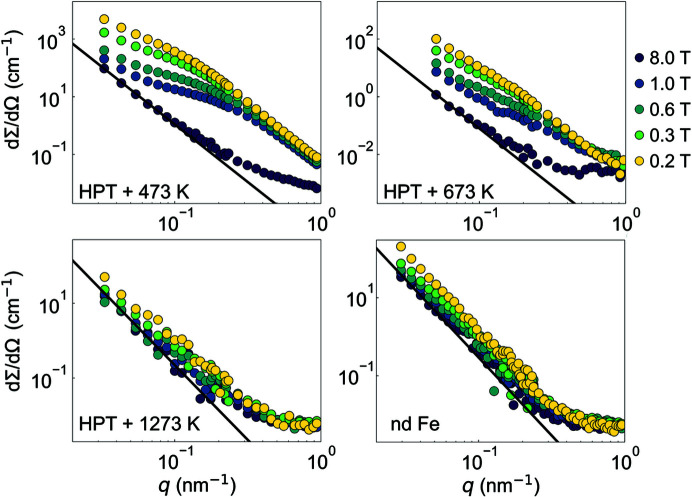
Magnetic field dependence of the (over 2π) azimuthally averaged total (nuclear + magnetic) SANS cross section dΣ/dΩ of HPT Fe and nd Fe (log–log scales). Black solid lines: asymptotic power law dΣ/dΩ ∝ *q* ^−4^.

Fig. 5 ▸ presents the magnetic-field dependence of the azimuthally averaged total SANS cross section dΣ/dΩ. As can be seen, at μ_0_ *H*_0_ = 8 T (near saturation), dΣ/dΩ can be well described by an asymptotic power-law exponent dΣ/dΩ ∝ *q* ^−4^ (at the smallest and intermediate *q* range), which is expected in the Porod regime and therefore suggests that dΣ/dΩ at 8 T is a good approximation of the residual SANS cross section dΣ_res_/dΩ. Compared with the cross section at 8 T, for HPT + 473 K and HPT + 673 K, the respective SANS profile at 1.0 T exhibits a (more or less pronounced) shoulder, similar to what has been reported in previous SANS studies of HPT ferromagnets (Oba *et al.*, 2020 ▸; Bersweiler *et al.*, 2021 ▸). With decreasing field, the scattering intensity increases by more than two orders of magnitude around the shoulder (*q* = 0.06 – 0.3 nm^−1^). Since the nuclear scattering is field-independent, the origin of the strong field dependence observed in HPT + 473 K and HPT + 673 K can be attributed to the spin misalignment, which is induced via HPT straining and persists in fields between 0.2 and 1.0 T. By contrast, for HPT + 1273 K and nd Fe, the *q* dependence of dΣ/dΩ becomes more similar to that obtained for dΣ_res_/dΩ ∝ *q* ^−4^ (at the smallest momentum transfers) and weakly field dependent. This suggests the presence of larger (nuclear + magnetic) correlation lengths in both samples (lying outside of the measured *q* range) and that the microstructures before deformation substantially reappear in HPT + 1273 K due to annealing. This hypothesis is supported by the rapid grain growth observed with increasing annealing temperature [see Fig. 2 ▸(*b*)].

**Figure 6:**
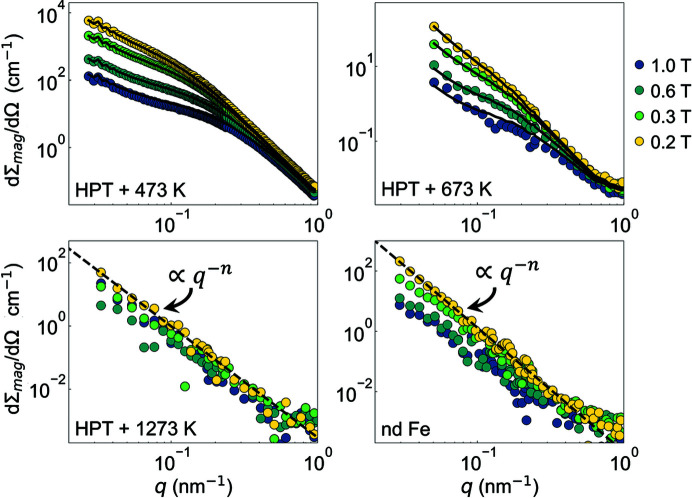
Magnetic field dependence of the (over 2π) azimuthally averaged purely magnetic SANS cross section dΣ_mag_/dΩ of HPT Fe and nd Fe (log–log scales). The dΣ_mag_/dΩ curves have been obtained from the data plotted in Fig. 5 ▸ by subtracting the dΣ/dΩ at 8.0 T from the dΣ/dΩ at lower fields. Black solid lines: fits by equation (5) using the scattering and response functions defined by equations (6)–(9), which are valid in the approach-to-saturation regime. Note that we have restricted our fit analysis to the HPT + 473 K and HPT + 673 K datasets, for which the magnetic correlation lengths can be spatially resolved within the available experimental *q* range [*i.e.* for neutron data which do not exhibit a power-law behavior of dΣ_mag_/dΩ ∝ *q* ^−*n*^ with *n* varying from ∼3.5 (4.1) to ∼3.9 (3.4) for HPT Fe (nd Fe)].

Fig. 6 ▸ displays the azimuthally averaged purely magnetic SANS cross section dΣ_mag_/dΩ. The magnitudes of the dΣ_mag_/dΩ are of the same order as the dΣ/dΩ shown in Fig. 5 ▸, therefore supporting the notion of dominant spin-misalignment scattering in HPT Fe. As previously suggested, it may originate from induced structural defects, which act as a source of effective magnetoelastic anisotropy field (Oba *et al.*, 2020 ▸). To estimate the anisotropy strength of the inhomogeneities, we have fitted the magnetic-field dependence of dΣ_mag_/dΩ according to equation (5), using the scattering and response functions given by equations (6)–(9) (solid lines in Fig. 6 ▸). More precisely, by taking the experimental value of the average saturation magnetization μ_0_ *M*_0_ = 2.1 T (of the HPT 473 K and HPT 673 K samples) and the estimated value of the exchange length *L*_0_ = 3.5 nm (assuming *A*_ex_ = 21 pJ m^−1^), only the global anisotropy factor *Q* is unknown in equations (6)–(9). The value for *L*_0_ (*A*_ex_) controls the global rate of decay with *q* of all the cross sections. Because of the linearity of equation (5) in *R*_H_ and *R*_M_, one can obtain the values of *S*_H_ and *S*_M_ at each *q* value by performing a (weighted) least-square fit of dΣ_mag_/dΩ measured at the different selected fields.^1^ The total least-square error of the fits was then numerically minimized to obtain the value of *Q*. The corresponding values for the global uniaxial anisotropy 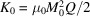 obtained for HPT + 473 K and HPT + 673 K are listed in Table 1 ▸. Note that the texture axis is assumed to be parallel to the incident neutron beam (*i.e.* along the HPT strain direction). As can be seen, the uniaxial anisotropy values obtained are slightly larger than the magnetocrystalline anisotropy value of 0.048 MJ m^−3^ reported for bulk Fe (Graham, 1958 ▸). Therefore, this result strongly supports the existence of an induced magnetoelastic anisotropy in pure Fe prepared by HPT straining, as previously suggested by Oba *et al.* (2020 ▸).

Moreover, as noticed in Fig. 6 ▸, the quality of the fits is reasonable with the largest deviations observable for the HPT + 673 K sample at around *q* = 0.15 nm^−1^. The (small) discrepancy between the experimental neutron data and the fits using equation (5) highlights the limits of the micromagnetic SANS theory for highly inhomogeneous ferromagnets (*i.e.* this study). More specifically, in the approach-to-saturation regime, the second-order micromagnetic SANS theory rigorously predicts that 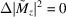 , so that the fourth term 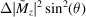 in equation (4) disappears (see Section 3). However, as already discussed in previous studies (Metlov & Michels, 2015 ▸; Metlov *et al.*, 2020 ▸; Oba *et al.*, 2021 ▸), it is entirely possible that for highly inhomogeneous ferromagnets, the higher-order terms become non-negligible, therefore breaking this property. Generally, the higher-order terms are masked by the lower-order terms. But, using unpolarized magnetic SANS, the field dependence of the higher-order contribution can be experimentally highlighted by considering the following combination of radially averaged SANS cross sections (Metlov & Michels, 2015 ▸):^2^


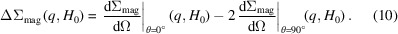

Inserting equations (5)–(9) into equation (10) shows that, for *Q* = 0, this combination of cross-section values is exactly zero (in second order). Non-zero ΔΣ_mag_ indicates the presence of higher-order scattering contributions.

**Figure 7:**
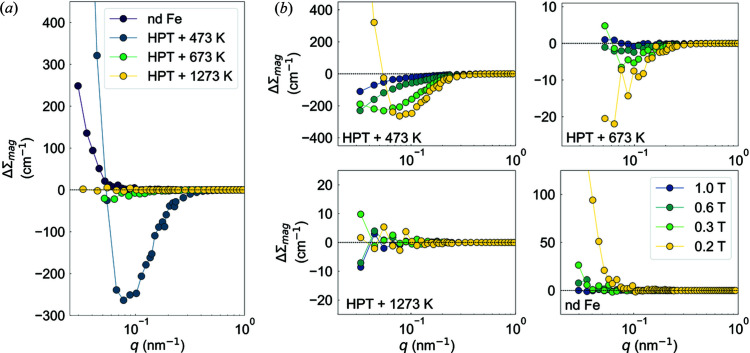
(*a*) Plots of the higher-order contribution ΔΣ_mag_ [equation (10)] at the selected field of 200 mT for HPT Fe and nd Fe. (*b*) Magnetic-field dependence of ΔΣ_mag_ for HPT Fe and nd Fe (semi-logarithmic scales).

Fig. 7 ▸(*a*) shows the higher-order scattering contribution ΔΣ_mag_ in HPT Fe and nd Fe at the selected field of 200 mT. As is seen, the magnitude and sign of ΔΣ_mag_ of these samples are strongly affected by the annealing temperature, which is further supported by the results for the field dependences of the individual ΔΣ_mag_ in Fig. 7 ▸(*b*). For HPT + 423 K and at 200 mT, ΔΣ_mag_ exhibits a local minimum at an intermediate *q* range, whereas for HPT + 673 K, ΔΣ_mag_ becomes strictly negative and small; for HPT + 1273 K, ΔΣ_mag_ ≃ 0. This result contrasts with the one obtained for nd Fe, for which ΔΣ_mag_ is strictly positive over the full *q* range. At the smallest momentum transfers and for the HPT + 423 K sample at 200 mT, the ratio of ΔΣ_mag_ to dΣ_mag_/dΩ is about 3−4%. The negative sign of ΔΣ_mag_ for HPT + 423 K can be qualitatively explained considering the micromagnetic SANS theory for a weakly inhomogeneous magnetic material (Metlov & Michels, 2015 ▸), which predicts that, in the case of anisotropy inhomogeneities with a very small amplitude, ΔΣ_mag_ may have negative values at an intermediate *q* range. Further extension of the higher-order effect theory is required to fully explain the strictly negative values of ΔΣ_mag_ over the whole *q* range in HPT + 673 K; a negative ΔΣ_mag_ was already reported for the case of HPT Ni (Oba *et al.*, 2021 ▸). Moreover, supported by the results of our structural characterization (XRD and EBSD), it appears clear that the variation in the magnitude of ΔΣ_mag_ in the HPT Fe samples is related to a change in their microstructure. Here, the reduction in the magnitude of ΔΣ_mag_ observed for the HPT Fe samples with increasing *T*_a_ can be attributed to a reduction in the density of defects that originate from the recovery and recrystallization process, which occurs at annealing temperatures larger than 473 K. Note that the shape of the inclusions (defects) might also play a significant role in the magnitude of ΔΣ_mag_. As theoretically explored by Schlömann (1971 ▸), for inhomogeneous magnetic materials, the higher-order contribution is found to be small for spherical inclusions and approximatively six times larger for layer-like inclusions. However, since the HPT process does not allow for the control of the shape of the grains in a material, this conjecture of Schlömann is difficult to verify or falsify. Fig. 7 ▸(*b*) displays the magnetic-field dependence of ΔΣ_mag_ in HPT Fe and nd Fe. As expected, the decrease of the magnitude of ΔΣ_mag_ with increasing *H*_0_ can be attributed to the fact that the transversal Fourier components 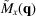 and 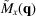 → 0 and that the longitudinal component 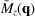 → 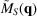 , which therefore implies 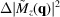 → 0 in equations (3) and (4).

## 5. Conclusions

We employed a combination of standard characterization techniques (XRD, EBSD, magnetometry) with a more advanced neutron method (magnetic SANS) to investigate the induced magnetoelastic anisotropy and the role played by the microstructure in the observation of higher-order scattering contributions in nanocrystalline Fe prepared by high-pressure torsion associated with a post-annealing process. The structural characterization confirms the formation of ultra-fine-grained pure Fe with different grain sizes by varying the subsequent annealing temperature. The fits of the neutron data to the micromagnetic SANS theory extended for textured ferromagnets yield anisotropy values of ∼0.059 and ∼0.073 MJ m^−3^ in ultra-fine-grained Fe with an average crystallite size of ∼48 and ∼98 nm, respectively. These values are larger than the magnetocrystalline anisotropy values for bulk Fe and strongly suggest the existence of an induced magnetoelastic anisotropy in ultra-fine-grained Fe prepared by HPT straining. Furthermore, the analysis of the magnetic field-dependent unpolarized SANS data unambiguously reveals the presence of non-negligible higher-order scattering contributions in the HPT + *T*_a_ samples. While the sign of the higher-order contribution might be related to the amplitude of the anisotropy inhomogeneities, as predicted by Metlov & Michels (2015 ▸), its magnitude appears clearly correlated to the changes in the microstructure (density and/or shape of the defects) induced by HPT and the subsequent annealing process. Finally, these results point out the necessity to consider the higher-order terms in the expression of the SANS cross section in the case of highly inhomogeneous ferromagnets.

## Figures and Tables

**Table 1:** Summary of the structural and magnetic parameters for HPT and non-deformed (nd) Fe determined by wide-angle XRD, EBSD and magnetometry

Parameters	nd Fe	HPT + 473K	HPT + 673K	HPT + 1273K
*D*_XRD_ (nm)	65 ± 5	48 ± 5	98 ± 5	77 ± 5
*D*_EBSD_ (µm)	∼722	∼0.51	∼3.6†	∼884
μ_0_ *M*_0_ (T)	2.06 ± 0.01	2.16 ± 0.01	2.04 ± 0.01	2.05 ± 0.01
*K*_0_ (MJ m^−3^)	–	∼0.059	∼0.073	–

† Note that Adachi *et al.* (2018 ▸) found a smaller grain size of 1.4 µm for this particular sample, which might be related to a lower statistical accuracy when evaluating the EBSD maps.

## Footnotes

1 Note that fitting the HPT Fe SANS cross section data with the response functions results in negligibly small values for *S*_M_. Therefore, for the fits in Fig. 6 ▸, we assumed that *S*_M_ = 0 exactly. Even with this reduced degree of freedom the fit quality is very good. This means that the magnetic scattering in our experiment is entirely due to the anisotropy constant fluctuations (contained in *S*_H_), while the saturation magnetization fluctuations (*S*_M_) are negligibly small. This can be considered further indirect evidence that our sample is single-phase and uniform (has no pores) on the SANS-relevant spatial length scale.

2 Typical examples for the 1D purely magnetic SANS cross section dΣ_mag_/dΩ along (θ = 0°) and perpendicular (θ = 90°) to *H*_0_ for the HPT + 473 K sample are shown in Fig. 4 ▸(*d*).
